# Direct comparisons of hand and mouth kinematics during grasping, feeding and fork-feeding actions

**DOI:** 10.3389/fnhum.2015.00580

**Published:** 2015-10-21

**Authors:** D. J. Quinlan, J. C. Culham

**Affiliations:** ^1^Brain and Mind Institute, University of Western OntarioLondon, ON, Canada; ^2^Department of Psychology, Huron University CollegeLondon, ON, Canada; ^3^Graduate Program in Neuroscience, University of Western OntarioLondon, ON, Canada; ^4^Department of Psychology, University of Western OntarioLondon, ON, Canada

**Keywords:** feeding, grasping, hand, mouth, fork, tool use, grip, transport

## Abstract

While a plethora of studies have examined the kinematics of human reach-to-grasp actions, few have investigated feeding, another ethologically important real-world action. Two seminal studies concluded that the kinematics of the mouth during feeding are comparable to those of the hand during grasping (Castiello, 1997; Churchill et al., 1999); however, feeding was done with a fork or spoon, not with the hand itself. Here, we directly compared grasping and feeding kinematics under equivalent conditions. Participants were presented with differently sized cubes of cheese (10-, 20- or 30-mm on each side) and asked to use the hand to grasp them or to use a fork to spear them and then bring them to the mouth to bite. We measured the apertures of the hand during grasping and the teeth during feeding, as well as reaching kinematics of the arm in both tasks. As in many past studies, we found that the hand oversized considerably larger (~11–27 mm) than the food item during grasping; moreover, the amount of oversizing scaled with food size. Surprisingly, regardless of whether the hand or fork was used to transport the food, the mouth oversized only slightly larger (~4–11 mm) than the food item during biting and the oversizing did not increase with food size. Total movement times were longer when using the fork compared to the hand, particularly when using the fork to bring food to the mouth. While reach velocity always peaked approximately halfway through the movement, relative to the reach the mouth opened more slowly than the hand, perhaps because less time was required for the smaller oversizing. Taken together, our results show that while many aspects of kinematics share some similarity between grasping and feeding, oversizing may reflect strategies unique to the hand vs. mouth (such as the need to have the digits approach the target surface perpendicularly for grip stability during lifting) and differences in the neural substrates of grasping and feeding.

## Introduction

Grasping and self-feeding actions are two of the most frequent everyday functions of the hand, particularly in humans and other primates (Graziano and Aflalo, 2007). In fact, such actions may be so fundamental in daily life that they shape the organization of the cerebral cortex (Graziano and Aflalo, 2007; Graziano, 2008). If regions of motor and premotor cortex are stimulated for a duration comparable to a natural action (e.g., a half-second), complex natural actions such as reach-to-grasp, self-feeding actions, or defensive actions can be evoked (Graziano et al., 2002). Moreover, different actions are evoked by stimulation to different foci and these foci are arranged topographically (Graziano et al., 2002). This topography has been observed across three primate species, suggesting it is common across the primate lineage (Kaas et al., 2013). Here, we compare the behavioral properties of two of these fundamental actions—grasping and feeding. Specifically, given that these two actions appear to have different neural substrates, we investigated whether their kinematic properties differ as well.

Although behavioral studies of feeding have been surprisingly few, a rich literature on the kinematics of reach-to-grasp actions has revealed the strategies employed in using the hand to acquire a target. The seminal studies of Jeannerod (1981, 1984, 1986) led to the proposal that reach-to-grasp actions are comprised of two distinct components: a transport component that uses visual information about object location to move the arm/hand to the target object and a grip component that uses visual information about intrinsic object properties such as shape and size to preshape the hand appropriately. Other evidence has suggested the transport and grip components may rely on different substreams of the dorsal visual pathway (Rizzolatti and Matelli, 2003; Cavina-Pratesi et al., 2010; Fattori et al., 2010; Vesia and Crawford, 2012; Turella and Lingnau, 2014). Hundreds of kinematic studies of reach-to-grasp movements have examined the factors that affect measures associated with transport and grip components (e.g., reach velocity and hand grip aperture, respectively; e.g., Jones and Lederman, 2006). However, feeding movements also involve arm transport (to the mouth) and aperture preshaping (by the mouth), but these components have been seldom investigated.

Two studies that have investigated self-feeding actions concluded that the transport and grip components of these reach-to-bite actions are similar to those of reach-to-grasp actions. One study measured kinematics while participants fed themselves cubes of cheese with a fork (Castiello, 1997). According to their description, participants “were required to reach for the cheese with the fork and bring it to the mouth” (p. 553) and “close[d] their mouths around the fork.” (p. 555). Results showed that as the cheese cube approached, the mouth opened to a size considerably larger than the cheese. This pattern is very similar to how the hand aperture oversizes and then closes down as the hand approaches the target object during a reach-to-grasp movement, as shown in previous data (Jeannerod, 1984). Similarities were also seen in the transport component, whereby the final approach took longer when the target object was small vs. large. Due to such similarities between reach-to-grasp and reach-to-bite kinematics, Castiello (1997) suggested that these actions might be directed by a common motor plan that is controlled by shared neural circuitry. In another self-feeding study (Churchill et al., 1999), participants fed themselves yoghurt using a spoon; similar results and conclusions were obtained as in Castiello (1997). Perhaps it is due to these proposed similarities between grasping and feeding actions that little subsequent research has been conducted on this topic.

**Figure 1:**
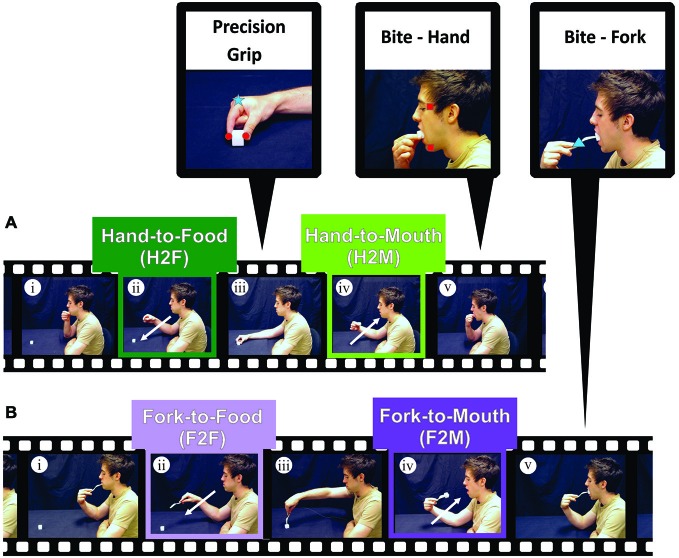
**Experimental procedure. (A)** Experiment 1. Participants began *Hand-to-Food* reaches with their right index finger and thumb in a closed pinch position, resting on their chin **(i)**. Participants then reached out toward a cube of cheese **(ii)** and grasped it with a precision grip **(iii)**. Once the food item was within the participant’s grasp, an inwardly directed reach toward the mouth (*Hand-to-Mouth*) was performed **(iv)**, ending the reach with a precision bite using the upper and lower incisor teeth **(v)**. **(B)** Experiment 2. Participants performed the same movement as **(A)** except that instead of using the index finger and thumb to capture and transport the food item, a fork was used to pierce the cheese and bring it to the mouth to bite **(i–v)**. Zoomed views at the top of the figure show the placement of markers (infrared-emitting diodes, IREDs) on the index finger and thumb (red circles; to measure hand aperture), on the temple and lower jaw (red squares; to measure mouth aperture), and on the hand or fork (blue square and blue triangle respectively; to measure transport kinematics).

While these studies of feeding actions were impressive initial forays into a new area of kinematic research, several aspects of the experiments may have artificially exaggerated the similarities between the kinematics of reach-to-bite and reach-to-grasp actions. First, the grip component was not comparable between the hand and mouth. That is, in grasping an object with a precision grip, the finger and thumb contact the sides of the object (Figure 1Aiii); whereas, in both of the feeding studies, the mouth was used not to grip the food (cheese cube or dollop of yoghurt) but to reach *around* the food and then pull it into the mouth. As such, in these feeding paradigms, the food served as an obstacle such that the mouth necessarily had to open larger than the food to avoid striking the teeth. Likewise, perhaps the presence of a “food obstacle” within the mouth grip aperture serves to inflate maximum aperture, much like the presence of obstacles outside the hand grip aperture cause peak aperture to become smaller (Jackson et al., 1995; Tresilian, 1998). Second, in Castiello’s (1997) study, the mouth aperture was determined by markers placed on the upper and lower lips. Alternatively, the markers could have been placed in a manner that would estimate the aperture between the teeth (or jaw). Although the aperture between the two lips would be correlated with the aperture between the teeth, the two are not always in perfect agreement because the lips are more elastic and can be moved somewhat independently of the teeth. In contrast, Churchill et al. (1999) placed markers on the forehead and chin, which would more directly reflect the aperture between the teeth because of skull and jaw anatomy. Notably, they found subtle kinematic differences between hand and mouth aperture. Lastly, both experiments had participants use a tool during the feeding actions, but compared the kinematics of the mouth to those exhibited when grasping with the hand alone. It remains a matter of debate how bodily actions are modified by tool use (e.g., Iriki et al., 1996; Cardinali et al., 2009; Gallivan et al., 2013). In fact, the introduction of a tool into reach-to-grasp actions alters some kinematic measures, such as lengthening the outward reach deceleration phase (Gentilucci et al., 2004). Taken together, these methodological differences between feeding and grasping may have affected the data and thus the conclusions; as such, it is worth re-examining how the two actions compare under conditions that are *as similar as possible*.

There are reasons to expect that reach-to-grasp and reach-to-bite actions may be performed differently when the two tasks are directly comparable. First, as mentioned there is evidence for different neural substrates (e.g., Buccino et al., 2001; Graziano et al., 2002). Second, reach-to-grasp and feeding actions may rely on different sensory information. In reach-to-grasp actions, the actor has clear vision of the object, the transport effector (arm), and aperture (hand) throughout the movement. In contrast, in feeding actions, the actor has clear vision of the object and transport effector (arm) initially but it degrades as the hand approaches the mouth [due to gaze (de Bruin et al., 2008) and limitations of vergence and accommodation]. Moreover, in feeding actions, the actor has no vision of the aperture (mouth).

Although visual information is much more limited for feeding than grasping actions, information from the somatic senses is richer and perhaps more highly weighted. During feeding actions, somatosensation provides additional information about the intrinsic object properties relevant for preshaping the mouth grip. Specifically, haptics and hand posture provide information about the size of an object as well as material properties such as its density and texture. Although proprioceptive and kinesthetic information about the arm’s location and trajectory is available for both grasping and feeding actions, some evidence suggests that inward arm movements may rely on proprioceptive information to a greater degree (de Bruin et al., 2008).

In sum, although two kinematic studies suggested strong similarities between grasping and feeding actions, other evidence suggests possible differences; as such we wanted to revisit the comparison of grasping and feeding kinematics under *directly comparable* conditions. In Experiment 1, participants reached out to grasp cheese cubes of three different sizes using a precision grip with the finger and thumb [i.e., *Hand-to-Food* (H2F) movement] and then brought the food to the mouth to perform a “precision bite” by gripping the cheese cube between the teeth [i.e., *Hand-to-Mouth* (H2M) movement]. We measured: (1) the grip component based on hand aperture during the H2F movement or mouth aperture during the H2M movement and (2) the transport component based the velocity of the arm during both H2F and H2M movements. In Experiment 2, we examined whether kinematics would be affected when participants used a fork, instead of their fingers, to acquire the food item [i.e., *Fork-to-Food* (F2F) movement] and bring the food item to the mouth [i.e., *Fork-to-Mouth* (F2M) movement]. We expected that our paradigm, with more directly comparable actions, may reveal differences between the kinematics of grasping vs. feeding movements.

## Materials and Methods

### Participants

#### Experiment 1

Ten right-handed participants (four males, six females; mean age = 31.1 years) with normal or corrected-to-normal vision participated in this experiment. Prior to testing, participants were required to undergo two prescreening tests: (1) handedness was assessed using a modified Edinburgh handedness inventory and (2) stereoscopic vision was tested with a 3-D Vectographs stereoacuity test (Stereo Optical Co., Inc., Chicago, IL, USA). Only those participants who were strongly right-handed and had normal depth perception were tested in the experimental paradigm. We also ensured that participants did not have allergies to dairy products (because the experiment involved cheese cubes) or adhesives (because the experiment involved mounting markers with spirit gum and medical tape). At the time Experiments 1 and 2 were conducted, all procedures were approved by the Department of Psychology Research Ethics Board (PREB) at the University of Western Ontario. The PREB was a sub-REB of The University of Western Ontario’s Research Ethics Board for Non-Medical Research Involving Human Subjects (NMREB) which was organized and operated according to the Tri-Council Policy Statement and the applicable laws and regulations of Ontario (Canada). Participants provided informed consent and were aware that they could terminate testing at any time.

#### Experiment 2

Ten right-handed participants (seven males, three females; mean age = 29.6 years) with normal or corrected-to-normal vision took part in this experiment. All participants met the same inclusion criteria as those used in Experiment 1 and had used cutlery (i.e., a fork) from an early age.

### Kinematic Data Collection

Participants’ movements were recorded by two three-camera opto-electronic recording systems (Optotrak, Northern Digital^TM^, Waterloo, Canada). These systems performed motion capture of the three-dimensional (3-D) positions of Infrared-Emitting Diodes (IREDs) attached to key locations on participants’ bodies. Using custom in-house software (OTCollect, programmed by Haitao Yang), the 3-D positions of each IRED were recorded at 100 Hz and used to calculate kinematic measures of transport and grip (e.g., reach velocity and aperture size). Each movement trial was recorded for a period of 3 s, enough time for the participant to reach out to grasp a food item and bring it to the mouth to bite it.

#### IRED Positioning

##### Experiment 1

Zoomed views in Figure 1 illustrate the placement of the IREDs used to track apertures and transport kinematics. IREDs used for calculating hand grip aperture were placed on the side of the distal thumb and index finger, such that when the thumb and finger were brought together in a “pinching” action, these IREDs were immediately adjacent. To measure reach velocity, an IRED was also placed on the side of the index finger knuckle (metacarpophalangeal joint), where the finger meets the hand. IREDs positioned on the hand were secured using cloth medical tape that did not perceptibly alter normal hand movement. To measure and calculate mouth aperture, IREDs were positioned on the left side of the chin and left temple (left mental tubercle of the mandible and sphenoid bone, respectively). Facial IREDs were secured in position using a spirit gum adhesive.

##### Experiment 2

As in Experiment 1, IREDs were positioned on the participant’s chin and temple to calculate mouth aperture measures. However, unlike Experiment 1, the participant’s reach velocity in Experiment 2 was determined by calculating the velocity of an IRED placed on the fork (See zoomed view in Figure 1). Previous studies investigating tool use in reach-to-grasp tasks have also employed similar IRED positioning (Churchill et al., 1999; Gentilucci et al., 2004).

### Procedure

#### Experiment 1

The focus of Experiment 1 was to investigate and compare the kinematics of Hand-to-Food and Hand-to-Mouth movements as performed during a natural feeding action without the use of tools (See Figure 1A). The names of the four-conditions performed in Experiments 1 and 2: (1) Hand-to-Food (H2F); (2) Hand-to-Mouth (H2M); (3) Fork-to-Food (F2F); and (4) Fork-to-Mouth (F2M)—are such that the first term of the condition name denotes what was used to capture and/or transport the food and the second term denotes the intended destination.

Prior to testing, participants were instructed to begin each trial in an “initial position” with the thumb and forefinger in the closed “pinch” position resting on the chin, with the mouth closed (upper and lower teeth touching) and with the eyes closed. A mozzarella cheese cube (10, 20, or 30 mm) was placed on the table at a comfortable reaching distance, approximately 30–40 cm away from the participant’s torso and along the body midline. Participants were then instructed to open their eyes and wait for an auditory beep (~ 200 ms) which signified the start of the trial (See Figure 1Ai). After this auditory cue, they were to simply reach out (Figure 1Aii), pick up the cheese cube with the thumb and forefinger using a precision grip (Figure 1Aiii) and bring the cheese cube to their mouth (Figure 1Aiv). Because we wanted participants to perform a mouth “grip” analogous to the hand grip during grasping, participants were instructed to bite the cube hard enough to hold the cube so that they could release their hand grip (Figure 1Av) but not to bite into the cheese. Upon completion of each trial, participants discarded the cheese cube, returned the hand to the initial position, closed their eyes, and waited for the next trial to begin. The absence of teeth marks on the cheese cubes verified that participants did not bite into the cheese. Participants were asked to perform this feeding action as naturally as possible and were given several practice trials prior to testing. The three cube sizes were presented in a randomized order until 15 repetitions of each size was completed. A fresh (unbitten) cheese cube was used on each trial.

#### Experiment 2

In Experiment 2, participants followed the same paradigm as in Experiment 1, except that instead of grasping the cheese cube with the thumb and forefinger, the cheese cube was skewered and transported to the mouth using a fork (See Figure 1Bi–v).

### Data Parsing

Custom in-house software (OTDisplay, programmed by Haitao Yang) was used to parse the movement data into to-Food and to-Mouth movements. As is typical of many reach-to-grasp kinematic studies, a reach velocity threshold of 20 mm/s was used to demarcate onset and offset of the outward reaches toward the food. If reach velocity did not drop below the 20 mm/s threshold between the outward and inward actions, the local minimum of the velocity trace was used as the offset of the outward reach and the onset of the inward reach. Because the mouth typically continued to close after the hand velocity dropped below this threshold in inward actions, the offset of the inward actions was defined as the point at which reach velocity had dropped below 20 mm/s and the mouth aperture ceased closing (i.e., velocity = 0 mm/s).

### Data Processing

Using the methods outlined below, the following dependent variables were calculated: (1) Oversizing (i.e., difference between maximum grip aperture and final grip aperture); (2) Time of Maximum Grip Aperture; (3) Total Movement Time; (4) Time of Peak Reach Velocity; and (5) Peak Reach Velocity. These computed values were then used in statistical analysis.

Hand grip aperture (i.e., the distance between the thumb and forefinger) was calculated from the vector distance between the thumb and index finger IRED coordinates and is generally accepted as a means of reporting grip aperture. However, as IREDs can only abut one another, there is an offset between these IREDs even during a closed pinch grip. This “offset” constant was subtracted from aperture measures so that the calculated vector distance was an accurate measure of the true distance between the gripping surface of the index finger and thumb.

Mouth grip aperture (i.e., the distance between the upper and lower incisor teeth) however, cannot be calculated in the same fashion because IREDs can not be placed directly on the teeth. Thus we chose to place IREDs on the chin and temple, positions that are not prone to occlusion or exaggeration of aperture. To calculate accurate mouth grip aperture, at the beginning/end of the testing session, participants performed calibration trials in which they bit hard plastic blocks of known size (i.e., 10, 20, 30, 40 and 50 mm). By plotting these known aperture values (i.e., block sizes) and the chin/temple IRED vector distances on an XY scatter-plot, we fit a third order polynomial function to these data points. We then used the function to convert the vector distance between chin and temple IREDs displayed during to-Mouth movements into accurate mouth aperture values.

Lastly, the IREDs on the forefinger knuckle (Experiment 1) and on the fork (Experiment 2) were used to calculate reach velocity during the outward and inward movements. Reach velocity was calculated from positional information from all three dimensions.

### Data Analysis

#### Extracting Data for Statistical Analysis

**Figure 2:**
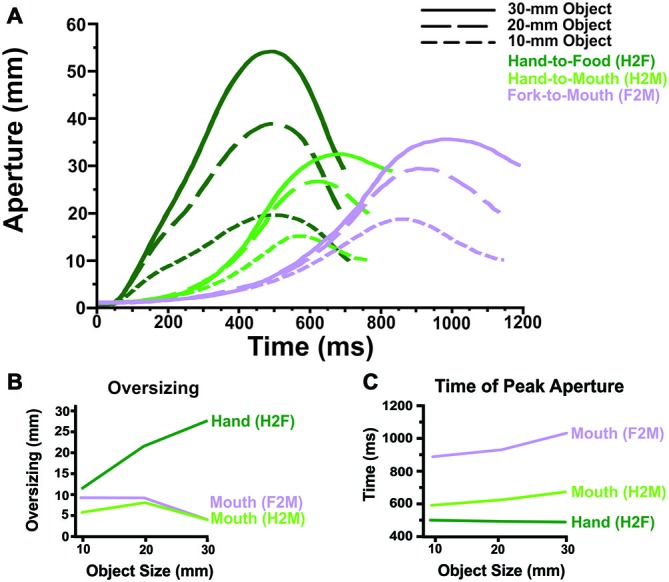
**Grip component results. (A)** Averaged aperture profiles (as a function of absolute time, in ms) illustrate that *Hand-to-Food*, *Hand-to-Mouth* and *Fork-to-Mouth* reaches display distinctly different grip patterns. **(B)** The hand oversizes considerably more than does the mouth, regardless of whether or not a fork was used to transport the food item. Moreover, oversizing scales with object size for the hand but not the mouth. **(C)** The mouth reaches peak aperture later than does the hand, particularly if a fork was used to transport the food.

**Figure 3:**
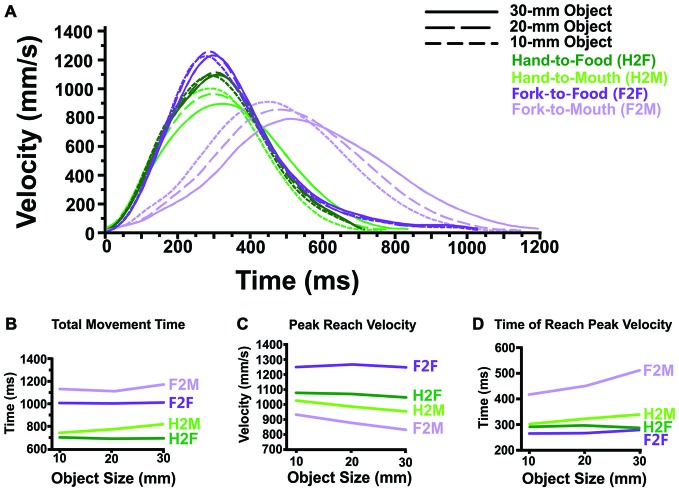
**Transport component results. (A)** Averaged reach velocity profiles (as a function of absolute time, in ms) illustrate that *Hand-to-Food*, *Hand-to-Mouth, Fork-to-Food* and *Fork-to-Mouth* reaches display distinctly different transport patterns. **(B)** Reaches toward the mouth (*Hand-to-Mouth* and *Fork-to-Mouth*) take longer than reaches toward food items (*Hand-to-Food* and *Fork-to-Food*). Likewise, reaches performed with a fork (*Fork-to-Food* and *Fork-to-Mouth*) take longer to execute than those reaches performed with the hand alone. **(C)** Reaches directed toward the mouth (*Hand-to-Mouth* and *Fork-to-Mouth*) attain lower peak reach velocities than do reaches directed toward food items (*Hand-to-Food* and *Fork-to-Food*), regardless of whether the reach is performed by the hand alone or a fork. Also, mouth-directed reaches also become slower as object size increases, a pattern not seen in food-directed reaches. **(D)** Fork reaches directed toward the mouth (*Fork-to-Mouth*) attain peak velocity far later than all other reach conditions. Similarly, reaches toward the mouth in general (*Hand-to-Mouth* and *Fork-to-Mouth*) attain peak velocity later than reaches directed toward food items (*Hand-to-Food* and *Fork-to-Food*). Also, *Fork-to-Food*, *Fork-to-Mouth* and *Hand-to-Mouth* reaches each attain peak reach velocity later as object size increases.

**Figure 4:**
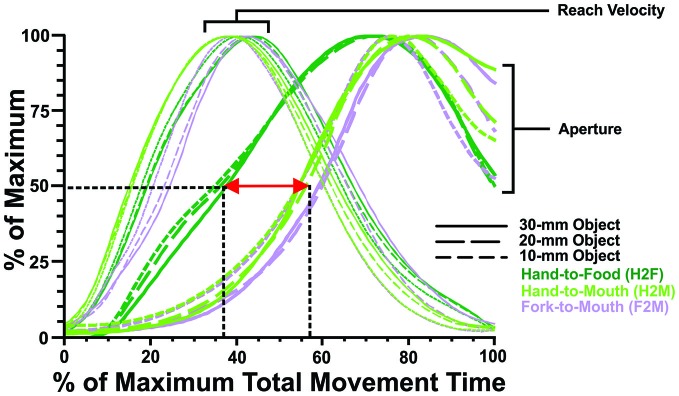
**Transport and grip component coordination.** To illustrate component coordination, the grip and transport measures for the H2F, H2M and F2M movements have been resampled to a duration of 100 time points (*x*-axis) and replotted as a percentage of maximal values on the *y*-axis. Reach velocity profiles are quite similar across testing conditions (H2F, H2M and F2M), reaching the peak 40–50% of the way through the movement; whereas aperture measures differ depending on reach direction (toward the food or toward the mouth). The aperture opens earlier for hand grasping than mouth biting, both when the aperture has reached 50% of its maximum (~37% vs. ~57% of movement time, respectively), and at its peak (~70% vs. ~80% of movement time, respectively).

All data and movement profiles for both Experiments 1 and 2 were first analyzed with absolute time (in ms) on the *x*-axis (rather than relative (%) time, as in some studies; Figures 2A, 2A, 3A). As each movement took a different amount of time to complete (even within the same condition), movement profiles for reach velocity and hand/mouth aperture in each of the experimental conditions needed to be averaged for each participant. To achieve these averaged profiles, the average number of time points it took a given participant to complete the movement of a given condition (Example: Subject A, 10 mm cube, H2F) was calculated. All the movement profiles for that particular condition were then resampled to the average number of time points it took that participant to complete that movement condition. This resampling method ensured that the value of a peak measure and the time at which it occurred were preserved to a greater degree than when trials within a given condition are simply averaged without resampling. This process was repeated for each of the experimental conditions and for each participant. Once this was completed, peak reach velocity, the time of peak reach velocity, aperture oversizing, and the time of peak aperture were extracted from the resampled movement profiles of each participant. These measures, along with total movement time, were then analyzed using a repeated-measures analysis of variance (ANOVA), followed by *post hoc*, paired-sample *t*-tests where appropriate. For qualitative comparisons of coordination between transport and grip components for the three conditions in which both transport and grip variables were available (H2F, H2M, F2M), the data were also replotted with the *x*-axis rescaled to relative (%) movement time and the *y-axis* (reach velocity for transport component; grip aperture for grip component) rescaled to a percentage of the maximum value (Figure 4).

#### Grip Component

Although a 2 (Effector: Hand vs. Fork) × 2 (Target: Food vs. Mouth) × 3 (Object Size: 10 vs. 20 vs. 30 mm) ANOVA is appropriate for transport-component measures, this is not possible for grip-component measures because during the F2F condition the hand already has a grip on the fork and therefore cannot provide hand aperture measures. Moreover, a 3 (Condition: Hand-to-Food vs. Hand-to-Mouth vs. Fork-to-Mouth) × 3 (Object Size: 10 vs. 20 vs. 30 mm) ANOVA is not appropriate for grip-component measures because one of the three conditions (F2M) involved a different sample of participants than the other two conditions (H2F and H2F). As such, we conducted three 2 (Conditions) × 3 (Object Size: 10 vs. 20 vs. 30 mm) ANOVAs to compare the following conditions and investigate possible interactions with object size: (1) hand vs. mouth aperture in H2F vs. H2M conditions (respectively; within-subjects repeated-measures ANOVA); (2) mouth aperture during H2M vs. F2M conditions (mixed-model ANOVA); and (3) hand vs. mouth aperture in H2F and F2M conditions (respectively; mixed-model ANOVA). The latter ANOVA was included because it enables comparison with the same contrast employed in earlier studies that compared grasping to fork-feeding (Castiello, 1997; Churchill et al., 1999).

Figure 2A illustrates how grip aperture changes as a function of time. The two dependent variables related to the grip component that were analyzed statistically (Oversizing and Time of Peak Aperture) are illustrated in Figures 2B,C.

#### Transport Component

All transport component measures (Total Movement Time, Peak Reach Velocity and Time of Peak Reach Velocity) were analyzed using a 2 (Effector: Hand vs. Fork, between-subjects) × 2 (Target: Food vs. Mouth, within-subjects) × 3 (Object Size: 10 vs. 20 vs. 30 mm, within-subjects) mixed ANOVA.

Figure 3A illustrates reach velocity as a function of time. The three dependent variables of the transport component are illustrated in Figures 3B–D.

## Results

One of the more salient features of Figures 2A, 2A, 3A is that the average durations of the movements across conditions are quite different. In many kinematic studies, where the movements performed do not differ as widely as those of the current experiments, it is commonplace to resample the movements to quantify kinematic measures of interest in terms of the percentage of movement time (Examples; Jeannerod, 1984; Marteniuk et al., 1990; Herbort and Butz, 2010). Here, we find that the differences in movement time across conditions (e.g., 500 ms) are so large that resampling would give a misleading impression of the temporal unfolding of the movements. Therefore, our primary analyses on the data (Figures 2, 2, 3) were computed on a real-time scale (in ms), which is later replotted on a relative-time scale (Figure 4) for comparisons between relative transport and grip timing.

### Aperture Oversizing

The most striking result for the aperture measures (See Figure 2B) was that the hand oversized much more during food grasping (H2F) than the mouth oversized during feeding with the hand (H2M) or a fork (F2M). In fact, the mouth typically only opened ~4–11 mm larger than the food while the hand typically opened ~11–27 mm larger. Furthermore, oversizing scaled strongly with object size for the hand, but not for the mouth. Mouth oversizing and its relationship to object size did not differ between feeding with the hand and feeding with a fork.

Statistical analyses supported these observations. There was a significant main effect of condition, main effect of object size, and interaction in the ANOVA comparing H2F vs. H2M × 3 sizes (all *p* < 0.001). Similarly, there was a significant main effect of condition, main effect of object size, and interaction in the ANOVA comparing H2F vs. F2M × 3 sizes (all *p* < 0.001). The ANOVA comparing mouth oversizing when feeding with the hand vs. fork (H2M vs. F2M) showed a main effect of object size (*p* < 0.001) but only a trend towards a main effect of condition (*p* = 0.06) and a trend toward an interaction (*p* = 0.06). *Post hoc* *t*-tests (Bonferroni-corrected *p* value of 0.05 for 18 comparisons = *p* < 0.0028) showed significantly greater oversizing for the hand than the mouth, regardless of whether the mouth was fed by the hand or a fork, at the two largest object sizes but not the smallest. In addition, *t*-tests showed that the hand oversized significantly more at larger sizes (20 vs. 10 mm, 30 vs. 20 mm, and 30 vs. 10 mm; *p* < 0.0028). In contrast, during H2M movements, the mouth showed less oversizing at the largest size (30 mm) than the middle size (20 mm), but no difference between the small size and the two larger sizes. Similarly, during F2M movements, the mouth showed less oversizing at the largest size (30 mm) than both of the smaller two sizes (10 and 20 mm; *p* < 0.001).

### Time of Peak Aperture

The results (See Figure 2C) showed that during grasping (H2F) the hand attains peak aperture significantly earlier than the mouth does when fed by hand or by fork (H2M and F2M). Also, mouth peak aperture occurs later when feeding with a fork (F2M) in comparison to feeding with the hand (H2M).

Statistical analyses supported these observations. There was a significant main effect of condition, main effect of object size, and interaction in the ANOVA comparing H2F vs. H2M × 3 object sizes (all *p* < 0.005). Similarly, there was a significant main effect of condition, main effect of object size, and interaction in the ANOVA comparing H2F vs. F2M × 3 object sizes (all *p* < 0.005). The ANOVA comparing time of peak mouth aperture when feeding with the hand vs. fork (H2M vs. F2M) showed a main effect of both object size and condition (*p* < 0.001), but no significant interaction. *Post hoc* *t*-tests (Bonferroni-corrected *p* value of 0.05 for 18 comparisons = *p* < 0.0028) showed that the hand reached peak aperture earlier than the mouth when feeding with the hand (H2F vs. H2M) for the two larger object sizes (20 and 30 mm; *p* < 0.0028) and approached our conservative significance level for the smallest object (10 mm; *p* = 0.006). Also, the mouth attained peak aperture later as object size increased when feeding by hand (H2M; 10 mm vs. 30 mm, *p* < 0.001), a pattern not present in hand aperture when reaching toward the food (H2F). In addition, the hand reached peak aperture (during hand feeding) earlier than the mouth when feeding with a fork (H2F vs. F2M) for all object sizes (*p* < 0.001). The main effects of Hand vs. Fork and Object Size during reaches to the mouth (H2M vs. F2M) demonstrate that the mouth reaches peak aperture later when a fork is used to feed oneself and that mouth reaches peak aperture later as object size increases.

### Total Movement Time

The two most notable patterns seen in total movement time (See Figure 3B) are that: (1) reaches made to the mouth (H2M and F2M) took longer to perform than those reaches directed toward the food (H2F and F2F) and (2) reaches with the fork (F2F and F2M) took longer than reaches performed with the hand (H2F and H2M).

Statistical analyses supported these observations. These effects were verified in the full (2 × 2 × 3) ANOVA, which showed significant main effects of Target (Food vs. Mouth; *p* < 0.005; within) and Effector (Hand vs. Fork reaches; *p* < 0.001; between) with no interaction between the two. Although there was no main effect of Object Size, there was a significant interaction between Target (Food vs. Mouth) and Object Size (*p* < 0.05) but no three-way interaction. To explore the Target × Object Size interaction, we collapsed across Effector and conducted *post hoc* *t*-tests (Bonferroni-corrected for nine comparisons, *p* < 0.0056) which only found a significant difference between outward movements toward the food vs. inward movements toward the mouth at 30 mm (*p* < 0.001).

### Peak Reach Velocity

One notable feature of Figure 3C is that reaches toward the mouth (H2M and F2M) display lower velocities than those reaches directed toward the food. Also, Peak Reach Velocity during Hand-to-Mouth and Fork-to-Mouth movements became slower as object size increased, whereas Hand-to-Food and Fork-to-Food reach velocity was unaffected by object size.

Statistical analyses supported these observations. The ANOVA revealed a significant main effect of both Target and Object Size (*p* < 0.005 and *p* < 0.001, respectively), a significant interactions of Effector × Target (*p* < 0.05), a significant interaction between Target × Object Size (*p* < 0.05) but no three-way interaction. To examine the Effector × Target interaction, we collapsed the data across Object Size, and performed *post hoc* *t*-tests (Bonferroni-corrected *p* value of 0.05 for four comparisons = *p* < 0.0125). These revealed that the interaction was driven by the fact that fork reaches directed toward the mouth were performed slower than fork reaches directed toward the food (*p* < 0.005), whereas a similar comparison of reaches performed with the hand alone failed to reach significance (*p* = 0.462). To examine the Target × Object Size interaction, we collapsed the data across Effector, and performed *post hoc* *t*-tests (Bonferroni-corrected *p* value of 0.05 for nine comparisons = *p* < 0.0056). These revealed that reaches toward the mouth were slower than those reaches directed toward the food at all object sizes (10 mm, *p* = 0.001; 20 and 30 mm, *p* < 0.001) and that reaches toward the mouth became slower as object size increased (10 mm vs. 20 mm and 10 mm vs. 30 mm, *p* < 0.001; 20 mm vs. 30 mm, *p* = 0.002).

### Total Distance Travelled

Note, however, an important caveat in interpreting peak velocity data. As visual inspection of Figure 3A shows, the total distance travelled (i.e., area under the curve) differed between conditions even though the physical distance between food and mouth remained constant.

First, although the total distance travelled was similar across sizes within a condition, it was longer for actions with the fork (43.0 cm) than actions with the hand (36.8 cm). IREDs used to record reach trajectories for hand and mouth were placed at similar distances from the fingertips or fork tip, respectively, and actions with both effectors required a ~180-degree rotation of the wrist. Nevertheless, the rotation arc was longer for the fork than the hand because the IRED was further from the wrist (pivot point).

Second and more interestingly, even within the same effector (and IRED), the total distance travelled differed for movements toward the food vs. toward the mouth. When using the hand, movements toward the food followed a longer path (H2F: 37.7 cm) than movements toward the mouth (H2M: 35.8 cm). We speculate that the hand may take more of an an arc trajectory en route to grasping the food (to ensure the index finger doesn’t hit the far edge of the cheese cube placed on the table) but more of a straight trajectory when delivering the food to the mouth. In contrast, when using the fork, the difference was reversed, with participants following a longer trajectory when bringing the fork to the mouth (43.8 cm) vs. the food (42.2 cm). We speculate that the fork does not need to follow an arc when stabbing the food (because it is aimed at the centre of the cube and doesn’t have to clear the edges) but may follow more of an arc when feeding such that the food approaches approximately perpendicular to the teeth. These speculations would be worth further investigation in future studies and would benefit from combining video recording of the actions in addition to kinematic tracking (e.g., Karl et al., 2012).

### Time of Peak Reach Velocity

One of the more notable features of Figure 3D is that fork reaches directed toward the mouth (F2M) attained peak velocity later than both fork reaches toward food (F2F) and hand reaches toward the mouth (H2M). It was also determined that reaches toward the mouth (H2M and F2M) attained peak velocity later than reaches directed toward the food (H2F and F2F). Also, there is evidence that both fork-reaches and reaches toward the mouth attain peak velocity later as object size increases.

Statistical analyses supported these observations. The ANOVA revealed significant main effects of Effector (*p* < 0.01), Target and Object Size (both *p* < 0.001). Moreover, there was a significant Effector × Target interaction (*p* < 0.001), a significant Target × Object Size interaction (*p* < 0.001), and a significant Effector × Object Size interaction (*p* < 0.05), but no three-way interaction. To investigate the Effector × Target interaction, we collapsed across Object Size and performed *post hoc* *t*-tests (Bonferroni-corrected *p* value of 0.05 for four comparisons = *p* < 0.0125). These revealed that reaches toward the mouth with a fork (F2M) attained peak reach velocity later than reaches toward the food with a fork (F2F, *p* < 0.001) and reaches toward the mouth with the hand (H2M, *p* < 0.001). There was no difference between reaches to the food vs. mouth when the hand alone was used (H2F and H2M). To investigate the Target × Object Size interaction (Bonferroni-corrected *p* value of 0.05 for nine comparisons = *p* < 0.0056), we collapsed across Effector and conducted *post hoc* *t*-tests. These revealed that reaches toward the mouth attained peak velocity later than reaches directed toward the food at all object sizes (*p* < 0.001). Also, there was some evidence to suggest that reaches toward the mouth attain peak velocity later as object size increases (10 mm vs. 30 mm, *p* < 0.005). Lastly, to investigate the Effector × Object Size interaction, we collapsed across Target and conducted *post hoc* *t*-tests (Bonferroni-corrected *p* value of 0.05 for nine comparisons = *p* < 0.0056). These revealed that fork-reaches attain peak velocity later as object size increases (10 mm vs. 30 mm, *p* < 0.005).

### Coordination of Transport and Grip Components

Although viewing the data in real time (that is, with ms on *x*-axis) gives the most accurate portrayal of how grasping and feeding actions unfold, it can also be valuable to examine the relative timing, which affords an easier comparison of how the transport and grip components of the actions are coordinated (cf. Churchill et al., 1999). Figure 4 shows the transport data (reach velocity) and grip data (aperture) replotted as a percentage of maximum over relative (%) time. These plots reveal that the transport component (reach velocity) unfolds quite consistently, with peak velocity achieved approximately 40% of the way through the movement. In contrast, the grip component (hand or mouth aperture) has a very different profile during grasping than feeding actions (regardless of whether feeding occurs using the hand or a fork). First, the hand aperture during grasping begins to open considerably earlier (with the hand aperture achieving 50% of maximum approximately 1/3 of the way through the movement) than the mouth does (with the mouth aperture achieving 50% of maximum over halfway through the movement). Second, the hand aperture peaks somewhat earlier (~70% of total movement time) than the mouth aperture (~80% of total movement time), though the timing differences appear less pronounced than in the earlier phases of aperture opening.

## Discussion

Our results demonstrate that when grasping and feeding movements are directly compared under highly similar conditions, the two actions clearly differ in the degree to which the hand and mouth oversize. Consistent with a large body of research on hand kinematics (beginning with Jeannerod, 1981, 1984, 1986), we found that the hand opens larger than the target during approach; moreover, maximum grip aperture scales with the size of the target. Surprisingly, however, we found that when the mouth directly bites the food items (cheese cubes in our case), oversizing is relatively small and nearly constant across object size. Although actions with a fork led to slower movements, particularly when the fork was brought to the mouth for feeding, the use of a fork had surprisingly little effect on mouth aperture.

The differences we observed between oversizing of the mouth and hand differ from the results of past investigations (Castiello, 1997; Churchill et al., 1999), which reported that the mouth aperture during feeding showed a similar degree of oversizing as the hand aperture shows during grasping. In these earlier studies, participants fed themselves with a fork (Castiello, 1997) or spoon (Churchill et al., 1999); however, the present results suggest the key difference between their results and ours was not the use of cutlery. That is, in our study, we found that the mouth showed little oversizing regardless of whether the hand or a fork was used to deliver the food. Rather, recall that in our study, participants bit the cheese cube *directly* with the teeth rather than pulling it off the implement into the mouth. We proposed that this approach makes the action of feeding have similar demands as grasping with the hand because both actions involve the closing of a bodily aperture upon—rather than around—the food item. In addition, we argue that our method of inferring the bite aperture (i.e., the aperture between the teeth) is more accurate than the previously used methods. Under these circumstances, we find far less oversizing of the mouth than the hand, particularly at large target sizes.

### Why does the Hand Oversize More than the Mouth?

The first and most obvious explanation for the greater oversizing of the hand than the mouth is simply that biomechanically the hand has a larger range of movement than the jaw; however, closer examination suggests a strong version of this explanation does not suffice. To investigate this hypothesis, we compared aperture oversizing displayed by the mouth, taking into consideration the maximum aperture the mouth is capable of producing. For the participants tested, the maximum aperture for the mouth was slightly larger than 50 mm (based on the largest block, 50-mm, used in calibration trials) so theoretically, participants could have reached a considerably larger maximum aperture for all sizes of the cheese cubes. Moreover, comparisons of apertures across the sizes argue against a hard limit. For example, in Figure 2A, the mouth reaches a maximum aperture of 36 mm when biting the largest (30-mm) object, yielding 6 mm of oversizing. Thus when biting the smallest (10-mm) object, theoretically the mouth could still open to 36 mm—but it doesn’t. Rather, the mouth opens only to a maximum of 19 mm, 9 mm larger than the food. Similarly, when biting the medium-sized object (20-mm), the mouth could open to a larger maximum aperture (36 mm) than it does (30 mm).

Note, however, that these arguments do not preclude weaker versions of a ceiling effect argument, including the possibility that participants minimized mouth oversizing because opening the mouth wider than strictly necessary may be relatively more uncomfortable than opening the hand wider than necessary. It also does not preclude the possibility that opening the mouth wider than strictly necessary may be considered impolite by one’s dinner companions (at least if they are adults). Interestingly, the fact that oversizing is less with the large (30-mm) cheese cube than the medium (20-mm) and small (10-mm) cheese cubes, may reflect “padded ceiling effect” (as suggested by a reviewer) in which the closer one gets to the limit the harder it pushes back. If indeed, this argument holds, it may partially account for why we see less oversizing (particularly for our 30-mm object) than Castiello did as he used smaller food items (5- and 20-mm cheese cubes).

A second possible explanation for the oversizing differences seen here is that *Hand-to-Food* and *Hand-to-Mouth* movements likely rely upon different sensory information for planning and adjustment. In particular, during grasping with the hand, visual information is available throughout the movement (including the visual preview to guide hand preshaping and visual feedback of the hand to enable online corrections) but haptic information is only provided following contact. In contrast, during feeding with the hand, an initial visual preview is available but visual feedback is limited (as the mouth is unseen, the view of the hand and food degrades during approach, and participants’ gaze does not follow the food; de Bruin et al., 2008) whereas haptic feedback about object size (from the hand) is available throughout the movement. Given that factors that increase uncertainty (e.g., removal of feedback) often lead to increased oversizing (Wing et al., 1986; Athènes and Wing, 1989), one possible explanation for the oversizing differences we observed is that participants had less uncertainty about object size during Hand-to-Mouth actions than Hand-to-Food actions. However, our fork-feeding results call this suggestion into question. That is, during fork-feeding, participants have less precise information about the food size (as it is no longer conveyed by hand aperture, though weight may still provide a partial cue), yet the maximum mouth aperture remained similar.

A third possible explanation could be that the mouth oversizes less due to a difference in the speed-accuracy trade-off between the goals. Put another way, when feeding, accuracy may be emphasized over speed to a greater degree than when grasping. Due to the slowed movement and increased accuracy, less oversizing may be needed.

A fourth possible explanation is that aperture closure strategies may differ between hand and mouth. Gripping an object is rarely the end goal of a hand grasping action; rather, typically it is the means to acquire an object for further manipulation such as lifting, moving, manually exploring—or even feeding. Indeed, participants show little or no oversizing when no such manipulation is possible, such as in flat pictures (Holmes and Heath, 2013) or grasps performed toward remembered objects that are no longer present (Goodale et al., 1994, Experiment 2). Manipulative actions require a firm grip to prevent slippage. One of the strategies proposed for achieving a stable precision grip is to have the index finger and thumb approach the target perpendicular to the respective surfaces at locations that transect the target’s centre of mass (Smeets and Brenner, 1999). This perpendicular approach strategy necessitates at least some grip oversizing which may unfold with a particular curvature to ensure smooth movements. In contrast, feeding actions such as our cheese biting task here serve different functions, typically to chew the food and/or bring the food to the tongue to initiate swallowing. Moreover, the effects of gravity may be more relevant for hand grasping, where slippage could lead to dropping the food, than mouth biting, where slippage is less consequential. As such, it may be that the benefits of oversizing are stronger for hand grasping to enable a smooth perpendicular approach of the digits than for biting. Consistent with this argument, others have found a similar absence of oversizing when participants grasped body parts on the face (Edwards et al., 2005), which are in no danger of slipping.

Of course, it may be that multiple factors, including all of the above, make some contribution to the kinematic differences between our conditions.

The relationships between aperture oversizing and object size are harder to interpret. Here, we found that hand oversizing increases with object size while mouth oversizing decreases (between the medium-sized and large objects). Note, however, that past studies of hand grip aperture as a function of object size have found mixed effects, with many finding a slope <1 (indicating that oversizing decreases with object size) with others finding a slope >1 (indicating that oversizing increases with object size (see meta-analysis in Smeets and Brenner, 1999, Figure 6A). Many studies do not use a careful calibration to examine oversizing *per se* but rather display the raw measurement of the distance between markers on the finger and thumb, which can include an offset. In addition, the effects could well depend on the range of sizes employed, with larger sizes being more likely to reveal hard or soft ceiling effects.

### Would the Mouth Always Show Less Oversizing than the Hand?

We have no doubt that many other potential variables could affect feeding strategies. These include the nature of the food and the typical means of feeding. Here, we used cubes of firm cheese (mozzarella) and instructed participants to bite the food without swallowing it. Our rationale for this was to make the biting action as similar as possible to the grip used in conventional grasping studies. However, we may have seen different outcomes for example if the subjects had been eating cubes of a softer cheese (e.g., brie) and simply using the teeth to move the food toward the tongue and throat or if we had used a harder cheese (e.g., parmesan) and instructed to take a bite. And of course there are many other foods (e.g., apples, popcorn) that have distinctive eating strategies. Our results also do not speak to the development of eating strategies. Anecdotally, infants open their mouths widely when being fed, although this may be due in part to the uncertainty of being fed by another person (Ferri et al., 2010). Nevertheless, our findings provide an interesting starting point for examining the kinematic strategies of feeding.

Interestingly feeding actions may affect not just the kinematics of the mouth but also the kinematics of the hand as it acquires the food during grasping. Specifically, when participants grasp a piece of food with the intention of placing it into the mouth, the maximum grip aperture of the hand does not open as wide as when they grasp the food with the intention of placing it in a bib below the mouth (Flindall and Gonzalez, 2013). This effect is only found with the right hand but not the left, which has led to the suggestion that the right hand is specialized for grasp-to-eat actions (Flindall and Gonzalez, 2013). Moreover, it occurs even if participants only bring the food to the mouth but do not actually eat it (Flindall and Gonzalez, 2014). Because our movement sequence involved grasping the food before bringing it to the mouth, these results suggest that the difference we observed between hand and mouth apertures would be even stronger if the grasping phase had involved a different goal (such as moving the cheese to a different location on the table).

### Neuroanatomy and Development of Grasping and Feeding Actions

In addition to behavioral differences, hand and mouth actions may rely on at least partially different neural substrates. For example, neurons in different divisions of premotor cortex respond to grasping vs. feeding actions (Rizzolatti et al., 1987, 1988). As detailed in the introduction, neurostimulation studies in other primate species (Graziano et al., 2002; Kaas et al., 2013) have revealed that hand grasping actions and feeding actions are evoked in different cortical sites within motor, premotor, and parietal cortex. These results suggest that both grasping and feeding (along with other actions like defensive movements and locomotion) may be ethologically relevant, fundamental actions within the motor repertoire that are associated with different properties (such as the region of space in which the actions occur or the reliance on different types of sensory information; Graziano and Aflalo, 2007).

While human neuroimaging has clearly identified the neural substrates for grasping and reaching actions (Binkofski et al., 1998; Culham et al., 2003; Castiello, 2005; Cavina-Pratesi et al., 2010; Turella and Lingnau, 2014), surprisingly little research has been done to investigate the neural substrates of feeding actions. In large part, this is due to technical limitations, especially with the predominant neuroimaging technique, functional MRI (fMRI). For example, our own attempts to study real feeding actions have been hampered by severe artifacts related to the movement of the mass of the arm (Barry et al., 2010), a larger problem for feeding actions (which recruit more proximal musculature: shoulders and biceps) than grasping (which can be performed predominantly using distal musculature: wrist and hand; Culham et al., 2003).

One early neuroimaging study used positron emission tomography (PET), which is not susceptible to mass motion artifacts, to examine human brain activation while participants grasped or bit a piece of candy off a fork moved toward the participant by the experimenter (Castiello et al., 2000). They reported similar activation for grasping and biting; however, the sample size was small (*n* = 5), the data were heavily smoothed (12-mm kernel), and the two actions were not directly contrasted. Thus this result suggests coarse similarity; however, it is possible that further investigation could reveal differences.

Indeed several fMRI studies have found that observation of actions with the hand and mouth (and in some cases other body parts) evoked activation in different, somatotopically organized foci within parietal, premotor, and lateral occipito-temporal cortex (Buccino et al., 2001; Wheaton et al., 2004; Pelphrey et al., 2005; Orlov et al., 2010). Specifically, while observation of hand grasping actions evoked activation in the anterior intraparietal sulcus and ventral premotor cortex, observation of mouth actions (such as biting an apple or chewing) yielded activation below these sites, in the anterior part of the inferior parietal lobule and inferior frontal gyrus, in or near Broca’s area (Buccino et al., 2001). In addition, while hand images activate the lateral occipitotemporal cortex (Bracci et al., 2010, 2012), mouth images activate a more anterior focus in the superior temporal sulcus (Wheaton et al., 2004; Pelphrey et al., 2005) and a more posterior/inferior focus (Orlov et al., 2010). The different neural substrates for hand actions and mouth actions raise the question of which foci would be activated by hand-to-mouth actions. One possibility is that such actions would evoke somatotopic activation for both effectors (hand and mouth); however, based on the neurostimulation studies in non-human primates (Graziano et al., 2002; Kaas et al., 2013), we expect that hand-to-mouth actions likely recruit different zones of sensorimotor cortex than hand-to-object actions like grasping.

Feeding and grasping movements may also differ in their developmental trajectories. Neonates are not only capable of making deliberate reaches to their mouth, they also make anticipatory mouth opening movements (Rochat et al., 1988; Blass et al., 1989; Takaya et al., 2003), suggesting that functional hand-mouth coordinated movements have developed prior to birth. Ultrasound movies of human fetuses have demonstrated that more than half of the arm movements produced (19–35 weeks gestation) resulted in hand contact with the mouth accompanied by anticipatory mouth opening, which suggests that these were intentional hand-mouth movements (Myowa-Yamakoshi and Takeshita, 2006). As little light is present *in utero*, it has been suggested that these movements are learned and performed using proprioception alone (Butterworth and Hopkins, 1988). In comparison, reaches to external targets are thought to develop rapidly over the first year of life and become fine-tuned throughout much of childhood (for review and longitudinal study, see Schneiberg et al., 2002), predominately guided by visual input; however, some propose that object-directed actions may initially rely more on proprioceptive/haptic guidance than visual guidance (e.g., Clifton et al., 1993; Karl and Whishaw, 2014).

### Timing and Coordination of Movements

In addition to differences in the magnitude of oversizing, clear differences were also observed in the timing of the movements. Most notably, feeding actions took longer than grasping actions, particularly when a fork was used and the relative coordination of aperture opening and reaching differed between grasping and feeding.

Taken together, these results suggest that the well-established temporal coordination between the transport and grip component differs for the hand and mouth. Perhaps because the mouth requires less oversizing, it can begin opening and reach peak aperture relatively later than the hand does because less time is required for closure.

One other notable difference between grasping and feeding actions is the combination of effectors involved. Grasping actions predominantly utilize arm, wrist and hand movements (as in most laboratory studies of grasping, objects are placed easily within reach and little torso movement is required). However, feeding actions require coordination of the arm, wrist and hand with the mouth, head and torso. During feeding, the actor may use trunk and head movements to a greater degree, especially when greater accuracy is required (e.g., taking a liquid vs. solid from a spoon; van der Kamp and Steenbergen, 1999).

### Conclusion

In conclusion, unlike previous studies of feeding actions which showed that grip and transport kinematics of grasping and feeding movements are similar, we show here that when equivalent movements of the hand and mouth are compared, numerous kinematic differences become apparent. In particular, when using their fingers to feed themselves, participants oversize the mouth considerably less than they oversize the hand when grasping. Although a number of explanations are possible, the one we favor is that grasping and biting may utilize different strategies. Moreover, they may rely on partially different neural substrates. The use of a fork to feed slowed the movement but had negligible impact on the grip component, including oversizing, suggesting that the key determinant of oversizing is the effector employed. Although these studies do not definitively explain the reasons for different strategies, they suggest that kinematically, and perhaps also neurally, feeding is not merely “grasping with the mouth” but rather has it own strategies worthy of further investigation.

## Author Contributions

DQ and JC conceived of the research and wrote the paper. DQ collected and analyzed the data.

## Conflict of Interest Statement

The authors declare that the research was conducted in the absence of any commercial or financial relationships that could be construed as a potential conflict of interest.

## Abbreviations

H2F: Hand-to-Food movement
H2M: Hand-to-Mouth movement
F2F: Fork-to-Food movement
F2M: Fork-to-Mouth movement.
